# Characterization of Somatostatin Receptor 2 Gene Expression and Immune Landscape in Sinonasal Malignancies

**DOI:** 10.3390/cancers16233931

**Published:** 2024-11-24

**Authors:** Elisabetta Xue, Dara Bracken-Clarke, Harris Krause, Tolulope Adeyelu, Mark G. Evans, Dilara Akbulut, Martha Quezado, Nishant Gandhi, Alex Farrell, Heloisa P. Soares, Emil Lou, Minh Phan, Rusha Patel, Ari M. Vanderwalde, Andrew Elliott, Conor E. Steuer, Nabil F. Saba, Daniel J. Lubin, Nyall R. London, James L. Gulley, Charalampos S. Floudas

**Affiliations:** 1 Center for Immuno-Oncology, National Cancer Institute, National Institute of Health, Bethesda, MD 20894, USA; elisabetta.xue@nih.gov (E.X.); dara.bracken-clarke@nih.gov (D.B.-C.);; 2 CARIS Life Sciences, Phoenix, AZ 85040, USA; 3 Laboratory of Pathology, National Cancer Institute, National Institute of Health, Bethesda, MD 20894, USA; 4 Huntsman Cancer Institute, University of Utah, Salt Lake City, UT 84112, USA; 5 Department of Medicine, Division of Hematology Oncology and Transplant, University of Minnesota, Minneapolis, MN 55414, USA; 6 Stephenson Cancer Center, University of Oklahoma, Oklahoma City, OK 73104, USA; 7 Department of Hematology-Oncology, Winship Cancer Institute of Emory University, Atlanta, GA 30308, USA; 8 Department of Otolaryngology-Head and Neck Surgery, Johns Hopkins University School of Medicine, Baltimore, MD 21218, USA; 9 Sinonasal and Skull Base Tumor Program, Surgical Oncology Program, Center for Cancer Research, National Cancer Institute, National Institute of Health, Bethesda, MD 20892, USA

**Keywords:** olfactory neuroblastoma, sinonasal undifferentiated carcinoma, sinonasal neuroendocrine carcinoma, somatostatin receptor 2, RNA-Seq, tumor microenvironment, exome sequencing, tumor biomarkers

## Abstract

Olfactory neuroblastoma (ONB), sinonasal undifferentiated carcinoma (SNUC), and sinonasal neuroendocrine carcinoma (SNEC) are rare malignancies arising from the sinonasal tract with limited therapeutic options. The expression of the somatostatin receptor 2 gene (*SSTR2*), which is expressed in other neuroendocrine neoplasms and is therapeutically actionable, has been reported in these tumors. Here, we analyzed *SSTR2* gene expression and its associations with genomic features, established biomarkers predicting of immune response, and the tumor immune microenvironment in a cohort of ONB, SNUC, and SNEC tumor samples (26, 13, and 8 samples, respectively) from a real-world database. *SSTR2* gene expression was high in neural-type ONB and low in basal-type ONB and in most of the SNUC and SNEC cases; there was no difference in expression between primary and metastatic tumors. The T cell-inflamed (TCI) score analysis classified 38.5% of SNUC cases as T cell-inflamed compared to only 3.9% of ONB and 0% of SNEC cases; 26.9% of ONB cases were classified as intermediate TCI; and SNEC had the lowest relative immune cell infiltration by deconvolution. In high *SSTR2*-expressing ONB, there was a higher proportion of infiltrating of Natural Killer cells and dendritic cells by deconvolution. Additionally, high *SSTR2*-expressing ONB was enriched for proliferation pathways, including E2F and Myc targets and G2M checkpoints. In conclusion, our findings delineate significant differences between these three types of sinonasal malignancies that were examined. In ONB, relative to SNUC and SNEC, the *SSTR2* expression profile, combined with its immune profiles, indicates potential novel therapeutic strategies and combinations for this unmet clinical need. Conversely, the inflammatory microenvironment of SNUC may be targetable using immuno-oncologic therapies.

## Simple Summary

Sinonasal carcinoma and olfactory neuroblastoma have very limited therapeutic options. The expression of the somatostatin receptor 2 (*SSTR2*) gene has been described in these patients, and the successful use of SSTR2-targeted treatments have been reported. This study investigates the association of *SSTR2* expression with genomic features, immune biomarkers, and the tumor immune microenvironment in a cohort of patients with sinonasal malignancies.

## 1. Introduction

Olfactory neuroblastoma (ONB), sinonasal neuroendocrine carcinoma (SNEC), and sinonasal undifferentiated carcinoma (SNUC) are rare sinonasal tumors with overlapping clinical, histopathological, and radiological findings. Prognosis varies according to both disease type and molecular features, with SNEC and SNUC showing more aggressive behavior compared to ONB. The primary treatment for localized ONB consists of surgery with the optional addition of adjuvant radiotherapy with or without chemotherapy; however, for recurrent or metastatic disease, the therapeutic arsenal is very limited. Treatment for SNUC consists of induction chemotherapy and chemoradiotherapy (CRT), with surgery being reserved for non-responders. Treatments for SNEC are less clearly defined and include both CRT and surgery depending on the extent of the disease and individual anatomy. Evidence for immune checkpoint inhibitors (CPIs) is presently only anecdotal with mixed results reported [1,2]. Furthermore, very few recurrent mutations have been identified in these malignancies, of which only *IDH2* is currently actionable and is currently under evaluation in a clinical trial (NCT06176989) [3,4]. The rarity of these tumors also represents a significant obstacle to conducting clinical trials; currently, no standardized strategies have been established for the advanced stages of these diseases.

Both SNEC and ONB are sinonasal tumors with varying degrees of neuroendocrine differentiation; SNEC includes both small-cell and large-cell neuroendocrine carcinomas, which have similar characteristics to their counterparts arising at other sites and are inherently high-grade and poorly differentiated. In contrast, SNUC is a diagnosis of exclusion and requires the absence of morphologic or immunohistochemical differentiation along a specific lineage (glandular, squamous, or neuroendocrine). However, while SNUC classically lacks the histological characteristics of neuroendocrine differentiation and is not classified as a neuroendocrine neoplasm, cases with neuroendocrine marker expression have been reported [5]. A recent study [6] examined the molecular features of these tumors in order to assist in diagnosis and tumor classification and reported similarities between them and classical neuroendocrine tumors. Additionally, two molecular subtypes of ONB with differences in the genomic and transcriptomic profiles and in the immune microenvironment have been described: basal (characterized by positivity for cytokeratin and an increased Ki67 index) and neural (enriched for the neuroendocrine markers chromogranin and synaptophysin) [7,8]. Furthermore, ONB was found to be clustered with glioblastomas, low-grade gliomas, paragangliomas, and pheochromocytomas following a Cancer Genome Atlas (TCGA) gene expression analysis of 29 cancers [8] and with low-grade gliomas, paragangliomas, and other glioneuronal tumors by DNA methylation using data from a larger cohort of central nervous system tumors [9]. A recent study further supports ONB transcriptional similarities with neuroendocrine cancers, particularly with small-cell lung cancer [6].

Somatostatin receptor 2 (SSTR2) is a transmembrane G-protein-coupled surface receptor widely expressed throughout the body, including but not limited to endocrine- and neuroendocrine-differentiated cells. It is widely expressed in well-differentiated neuroendocrine tumors (NETs) and is actionable both diagnostically and therapeutically. Metabolic imaging with positron emission tomography (PET) integrated with computed tomography (CT) using radiolabeled SSTR2 ligands (gallium-68 DOTATATE and DOTANOC) is, in general, used as the international standard for the staging and follow-up of NETs [10,11]. Somatostatin receptor 2 has also been successfully targeted therapeutically, both directly using somatostatin analogues (e.g., octreotide and lanreotide) and using radionuclide therapy (peptide-receptor radionuclide therapy—PRRT) consisting of a somatostatin analogue conjugated to a radioisotope (e.g., ^177^Lutetium) [12]. Cellular therapies and antibody–drug conjugates targeting SSTR2 are currently in development [13,14].

The expression of *SSTR2* in ONB and SNEC has been reported across tumor grades and in both primary and metastatic sites [15,16]; its expression in SNUC appears to be less clear [17,18]. Somatostatin receptor 2-directed imaging with ^68^Ga DOTATATE PET/CT has been suggested as an alternative to ^18^F-Fluorodeoxyglucose-PET in sinonasal malignancies as it allows for better tumor characterization due to the absence of brain uptake [19]. Somatostatin receptor 2-directed therapeutic approaches have also been tested in small ONB and SNEC cohorts with encouraging results [20,21]. We studied *SSTR2* gene expression and its association with genomic features, immune biomarkers, and tumor immune microenvironment composition in a cohort of ONB, SNEC, and SNUC.

## 2. Patients and Methods

### 2.1. Specimens

We studied the molecular profiles of ONB, SNEC, and SNUC tumor specimens that underwent next-generation sequencing (NGS) of DNA (592-gene panel or whole-exome sequencing [WES]) and whole-transcriptome sequencing (WTS) at a Clinical Laboratory Improvement Amendments (CLIA)-certified clinical laboratory (Caris Life Sciences, Phoenix, Arizona, USA). Specimens were classified as metastatic or primary based on their site of biopsy. This study was performed in accordance with the guidelines of the Declaration of Helsinki, the Belmont Report, and US Common Rule. In compliance with policy 45 CFR 46.101(b), this study was conducted using retrospective, de-identified clinical data, and patient consent was not required.

### 2.2. Cohort Characteristics

We analyzed data from 47 tumor specimens across the 3 tumor types: 26 ONB, 13 SNUC, and 8 SNEC cases. Patient and tumor characteristics are summarized in Table 1. No information on tumor grade was available for review.

### 2.3. DNA Next-Generation Sequencing (NGS)

A targeted 592-gene panel or WES was performed using genomic DNA isolated from micro-dissected formalin-fixed paraffin-embedded (FFPE) tumor specimens. The 592-gene panel was sequenced using the NextSeq platform (Illumina, Inc., San Diego, CA, USA). A custom-designed SureSelect XT assay was used to enrich 592 whole-gene targets (Agilent Technologies, Santa Clara, CA, USA). The Illumina NovaSeq 6000 sequencer (Illumina, Inc.) was used to perform WES. A hybrid pull-down panel of baits designed to enrich for 700 clinically relevant genes at a high coverage and a high read depth was used, along with another panel designed to enrich for an additional >20,000 genes at a lower depth. Five hundred and ninety-two gene and WES assays were cross-validated and demonstrated highly concordant results. Matched normal tissue was not sequenced.

#### Identification of Genetic Variants

Genetic variants identified were categorized as ‘pathogenic’, ‘likely pathogenic’, ‘variant of unknown significance’, ‘likely benign’, or ‘benign’ according to the American College of Medical Genetics and Genomics (ACMG) standards; ‘pathogenic’, and ‘likely pathogenic’ variants were counted as mutations, while ‘benign’ variants, ‘likely benign’ variants, and ‘variants of unknown significance’ were excluded when assessing mutation frequencies of individual genes.

### 2.4. RNA Whole-Transcriptome Sequencing (WTS)

In order to determine the percentage of tumor content and tumor size, FFPE specimens underwent pathology review; a minimum of 10% of the tumor content in the area for microdissection was required to enable the extraction of tumor-specific RNA. A Qiagen RNA FFPE kit (Qiagen, Germantown, MD, USA) was used, and the RNA quality and quantity were determined using the Agilent TapeStation (Agilent Technologies, Santa Clara, CA, USA). Biotinylated RNA baits were hybridized to the synthesized and purified cDNA targets, and the bait–target complexes were amplified in a post capture PCR reaction. The resultant libraries were quantified and normalized, and the pooled libraries were denatured, diluted, and sequenced. For transcript counting, transcripts per million (TPM) molecules were generated using the Salmon expression pipeline.

For comparison purposes, transcriptional profiles of gastrointestinal neuroendocrine tumors (GI-NETs) selected from the same real-world database using the Oncotree classification [22] were used.

To study transcriptional differences between high and low *SSTR2*-expressing tumors, differential expression analysis was performed using PyDeseq2 [23]. Gene Set Enrichment Analysis (GSEA) was performed on the WTS data using the Hallmark gene set collection from the Human Molecular Signature Database [24,25].

### 2.5. Immunotherapy-Related Biomarkers, Signatures, and Immune Cell Infiltration

We assessed tumor mutational burden (TMB) through WES; high tumor mutational burden (TMB-H) was defined as ≥10 mutations per megabase. Tumor microsatellite instability-high (MSI-H) status was determined by NGS assessment (7000 target microsatellite loci were examined and compared to the reference genome hg19 from the University of California, Santa Cruz), whereas mismatch repair-deficient status (dMMR) was determined by immunohistochemistry (IHC) for MLH1 (M1 antibody), MSH2 (antibody G2191129), MSH6 (antibody 44), and PMS2 (antibody EPR3947, Ventana Medical Systems, Inc., Tucson, AZ, USA). The two methods generated highly concordant results; for the rare discordant cases, the dMMR status by IHC was prioritized over MSI-H status by NGS. Programmed Cell Death Ligand 1 (PD-L1) expression was assessed by IHC using 22C3 antibody staining (IHC-PD-L1 22C3), and PD-L1 positivity was defined as a tumor proportion score (TPS) ≥ 1%.

Tumor immune cell infiltration was estimated using quanTIseq, a computational deconvolution method that uses RNA transcripts known to be expressed in specific immune cell types to estimate the different immune cell fractions from tumor-derived bulk RNA sequencing data [26]. Transcriptomic data were utilized to calculate the T cell-inflamed (TCI) score, a transcriptional signature predictive of the immunotherapy response, with a score < −80 being categorized as not T cell-inflamed, a score ≥ 80 as T cell-inflamed, and the rest being intermediate [27].

### 2.6. Epstein–Barr Virus and Human Papillomavirus Status

Samples in our cohort were tested for EBV and HPV-16/18. Sinonasal malignancies have rarely previously been reported as being positive for Epstein–Barr virus (EBV) and/or Human Papillomavirus (HPV) [28]; thus, EBV and HPV statuses were assessed. Epstein–Barr virus status was defined by EBV-encoded small RNAs (EBER), in situ hybridization (ISH), or WES with EBER ISH performed on FFPE sections on glass slides. Slides were stained using automated staining techniques per the manufacturer’s instructions and were optimized and validated per CLIA, the College of American Pathologists (CAP), and the International Organization for Standardization (ISO) requirements with the EBER status being determined by a trained pathologist. Epstein–Barr viral status was also evaluated by WES using ≥25,000 EBV DNA read counts as a threshold to determine EBV positivity. When both EBER ISH and EBV detection were performed using WES, results were concordant in all cases. When EBER ISH was available, slides were examined to determine the exact localization of EBV within the tumor samples. Digital images of hematoxylin and eosin (H&E) slides were reviewed by three pathologists to review the primary diagnosis of EBV^pos^ SNUC. HPV16/18 status was detected using 39 unique baits to detect HPV16 and 50 unique baits to detect HPV18 out of a total of 2360 total pathogen baits. The threshold for positivity was considered ≥300 reads for either HPV16 or HPV18.

In our cohort, all ONB and SNEC samples were negative for both EBV and HPV, whereas among SNUC samples, one was HPV^pos^ and one was EBV^pos^ as determined by EBER ISH. Pathology review of the EBV^pos^ SNUC sample confirmed SNUC diagnosis with lack of morphologic differentiation, such as glandular, squamous, or neuroendocrine features; EBER ISH positivity was localized to the tumor cells.

### 2.7. Statistical Analysis

Statistical analyses included the Mann–Whitney U, Fisher’s Exact, and χ^2^ tests as appropriate, with *p*-values being adjusted for multiple comparisons (significance set at *p* < 0.05). Real-world overall survival information was obtained from insurance claims data and calculated from time of specimen collection to last follow-up. Kaplan–Meier estimates were calculated for the molecularly defined patient cohorts. *p*-values were calculated using the log-rank test with significance determined as a *p*-value of <0.05.

## 3. Results

### 3.1. SSTR2 Expression

**Figure 1:**
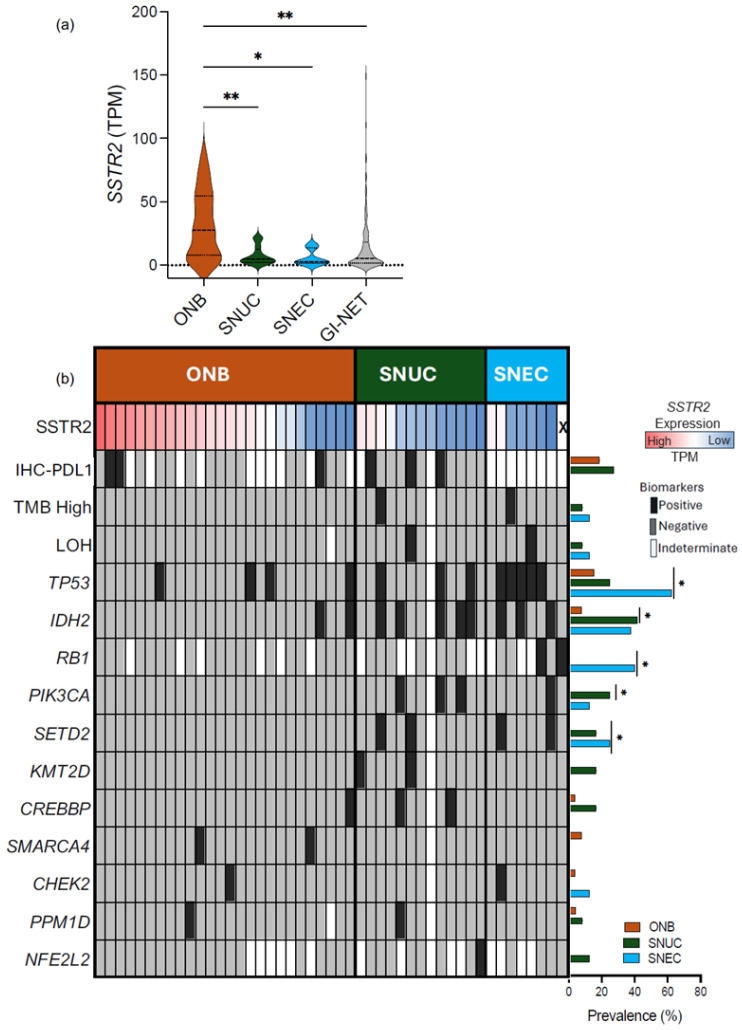
(**a**) SSTR2 expression in transcripts per million (TPM) for the indicated tumor types (asterisks indicate statistical significance; * *p* < 0.05 and ** *p* < 0.01). (**b**) Oncoprint showing the prevalence of immune biomarkers (IHC-PD-L1 measured through a 22C3 antibody assay; TMB—high classification) and the prevalence of pathogenic mutations (pathogenic single-nucleotide variant/insertion–deletion) for each tumor type. Asterisks indicate statistical significance; * *p* < 0.05.

Transcripts of *SSTR2* were detected in all ONB samples (median of 27.9 TPM; range of 1.11–87.18), all SNUC samples (median of 4.7 TPM; range of 0.85–22.03), and all SNEC samples (median of 3.1 TPM; range of 0.44–16.49), as presented in Figure 1a; *SSTR2* expression in GI-NETs was included as a comparator (N = 153; median of 5.53 TPM; range of 0.05–46.36). The expression of *SSTR2* was significantly higher in ONB compared to SNUC, SNEC, and GI-NETs. There was no statistically significant difference in *SSTR2* expression between the primary and metastatic samples in either the gastrointestinal NETs or ONB (data not shown), and given the small sample size, no analysis could be performed for primary vs. metastatic SNUC and SNEC.

### 3.2. Genomic Alterations

We examined the tumor genomic landscape of ONB, SNUC, and SNEC (Figure 1b). Olfactory neuroblastoma was enriched for *TP53* mutations, present in 4/26 (15.4%), followed by *IDH2* and *SMARCA4* mutations, both present in 2/26 (7.7%). Sinonasal undifferentiated carcinoma was enriched for *IDH2mut*, identified in 5 of 12 (41.7%) samples, followed by *TP53mut* and *PIK3CAmut*, both present in 3 of 12 (25%) samples. The most frequently mutated gene in SNEC was *TP53* (5/8, 62.5%), followed by *RB1* (2/5, 40%) and *IDH2* (3/8, 37.5%); *ARID1A* mutations were not identified. There was no association between high *SSTR2*-expressing tumors and specific genetic mutations in ONB, SNUC, or SNEC.

### 3.3. Immune Features

Programmed death ligand-1 staining (Figure 1b) was available for 16/26 (61.5%) of ONB cases and was positive (TPS ≥ 1.0%) in 3/16 (18.8%) of cases. Similarly, 3 out of 11 (27.3%) SNUC samples tested for PD-L1 were positive according to the TPS. No data on PD-L1 staining were available for SNEC. None of the ONB cases, 1 out of 11 SNUC cases, and 1 out of 8 SNEC cases were classified as TMB-H. None of the ONB, SNUC, nor SNEC specimens were positive for dMMR/MSI-H status. In ONB, two out of three specimens with a TPS ≥ 1.0% showed high *SSTR2* expression; no association between specific genomic mutation and *SSTR2* expression was seen. Of note, both *IDH2* mutated specimens showed low *SSTR2* expression.

**Figure 2:**
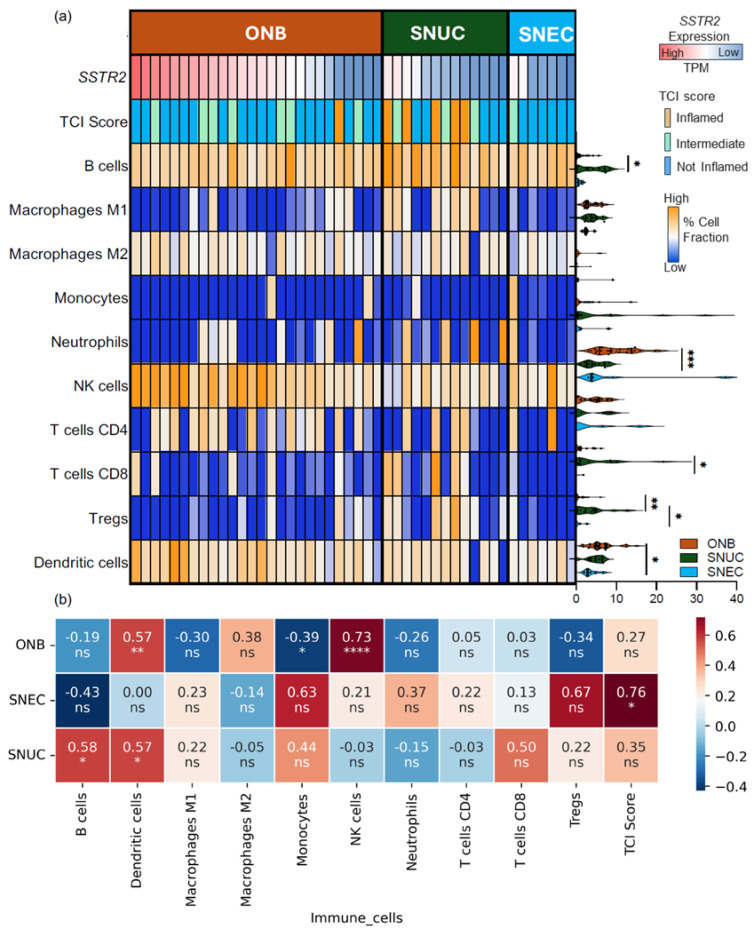
(**a**) Heatmap showing SSTR2 expression (transcripts per million: TPM), prevalence of T cell-inflamed subtypes, prevalence of immune biomarkers, and % immune infiltrate (derived from bulk RNA sequencing using quantTIseq) for indicated tumor types. (**b**) Heatmap showing correlation of immune cell fractions and T cell-inflamed score with *SSTR2* expression level. Correlation and corresponding *p*-values are shown within heatmap. Asterisk indicates statistical significance; * *p* < 0.05, ** *p* < 0.01, *** *p* < 0.001, and **** *p* < 0.0001. Abbreviations: SSTR2—somatostatin receptor 2; TCI score—T cell-inflamed score; ns—non significant.

We estimated the tumor immune cell composition across the studied tumor types using quanTIseq, an RNA-based immune deconvolution method (Figure 2a,b and Table S1): SNEC had the lowest rate of immune cell infiltration at 18.6% compared with SNUC (29.2%) and ONB (28.5%). B cells were the most frequent immune cell population estimated in both SNUC and SNEC cases (median B cell fractions of 9.2% and 8.76%, respectively), whereas Natural Killer (NK) cells (median NK cell fraction of 8.60%) were the predominant estimated immune cell population in ONB.

Given the variable expression of *SSTR2* in ONB, samples were discretized into high and low *SSTR2*-expressing subgroups based on median expression (27.9 TPM), with the goal of evaluating the impact of *SSTR2* on the tumor microenvironment. The high *SSTR2*-expressing ONB cases had a greater estimated proportion of NK cells (median 13.0% vs. 6.3%; *p* = 0.006) and dendritic cells (7.2% vs. 5.0%; *p* = 0.02) compared to the low-*SSTR2* ONB cases, with no difference being found in the estimated proportion of T cells, B cells, and macrophages (Figure S1 and Table S2). In SNUC, higher *SSTR2* expression correlated with increased B and dendritic cells, whereas in SNEC, no correlation with immune cell composition was observed, potentially due to the overall low *SSTR2* expression (Figure 2b).

Tumor inflammation characterized by the TCI transcriptomic score was analyzed for these tumors (Figure 2a,b): in ONB, the median TCI score [26] was −93.0 (interquartile range, IQR: −106.5–−67.8), with only 1/26 (3.9%) tumors being classified as T cell-inflamed, 18/26 (69.2%) tumors as non-inflamed, and 7/26 (26.9%) as intermediate. The median TCI score for SNUC was –1.5 (IQR: −113.5–3.0), with 5/13 (38.5%) being classified as T cell-inflamed, 5/13 (38.5%) as non-inflamed, and 3/13 (23.1%) as intermediate. In the case of SNEC, the median TCI score was −121.1 (IQR: −149.5–(−133.5)); no tumor was classified as inflamed, seven (87.5%) were classified as non-inflamed tumors, and one (14.3%) as intermediate. The expression of *SSTR2* did not correlate with the TCI score for ONB nor SNUC, and for SNEC, the only sample with an intermediate TCI also showed high *SSTR2* expression.

### 3.4. Transcriptomic Differences in High vs. Low SSTR2-Expressing ONB

**Figure 3:**
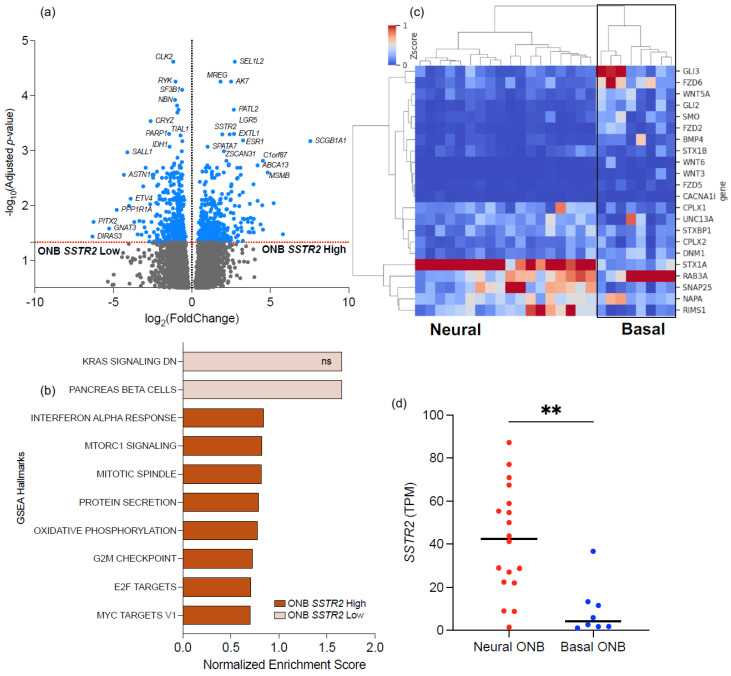
(**a**) Differentially expressed genes between high *SSTR*-expressing ONB vs. low SSTR2-expressing ONB. (**b**) Gene set enrichment analysis between high vs. low SSTR2-expressing ONB. (**c**) Heatmap depicting neural- and basal-associated genes. (**d**) *SSTR2* expression compared between neural and basal ONB. Asterisks indicate statistical significance; ** *p* < 0.01. Abbreviations: ns, non statistically significant.

Differential gene expression was studied between high vs. low *SSTR2*-expressing ONB, and a GSEA was performed (Figure 3a,b). This revealed an enrichment of several hallmark pathways, such as E2F targets, the G2M Checkpoint, Interferon Alpha Response, and MYC Targets V2, among others upregulated in the high *SSTR2*-expressing ONB group; similarly, the ‘KRAS signaling down’ pathway was enriched in the low *SSTR2*-expressing ONB group.

A transcriptomic analysis [8] suggested that ONB can be divided into neural and basal subtypes, with neural ONB demonstrating an expression profile associated with neural differentiation genes and basal ONB demonstrating a more undifferentiated profile, overexpressing cytokeratin. We clustered our ONB cohort in basal and neural subtypes using Ward’s clustering method (Figure 3c,d and Figure S2); despite great variability in *SSTR2* expression being noted in the neural subtype, we observed significantly higher *SSTR2* expression in the neural (median 42.5 TPM) compared to the basal subtype (4.2 TPM; *p* = 0.002).

No difference in overall survival was seen between patients with high vs. low SSTR2-expressing ONB, although a trend towards improved survival in the former group was observed (Figure S3).

## 4. Discussion

We characterized *SSTR2* expression in ONB, SNUC, and SNEC; examined its associations with genomic alterations and immune biomarkers; and estimated immune cell infiltrates. *SSTR2* was expressed across ONB subtypes (albeit at different levels) and correlated with variations in the microenvironment composition. Relatively lower *SSTR2* expression was seen in SNUC and SNEC.

In this dataset, the genomic profiling of ONB revealed a lack of targetable mutations, with only 7.7% of samples (2 of 26) harboring *IDH2* mutations; ONB samples appeared to have a low estimated proportion of infiltrating T cells, with almost 70% showing a low TCI score, suggesting an immunologically cold microenvironment in most cases [4]. Only 18.8% of samples in this cohort were PD-L1 positive (TPS ≥ 1%), which is within the range reported in previous studies (PD-L1+: 7% to 40%) using varying scoring methodologies and derived from small cohorts [29]. The results of transcriptomic deconvolution revealed a higher estimated proportion of NK cells compared to what has previously been reported [30,31,32]. In a recent report of 45 tumor types not including sinonasal malignancies [33], an analysis of NK cell infiltration with the same transcriptomic deconvolution method that was used in the present analysis showed that low-grade gliomas and glioblastomas were among the tumors with the highest NK cell fraction. Given the reported similarity in transcriptomic profiles reported between ONB, glioma, and glioblastoma [8], the concordance in NK cell infiltration inferred from RNA sequencing with RNA deconvolution methods may not be unexpected.

In our cohort, there was significantly higher *SSTR2* expression in ONB compared to non-ONB sinonasal cancers, especially for neural ONB [34,35], which supports a greater degree of neuroendocrine differentiation in this subtype [8]. Decreases in *SSTR2* expression with an advancing stage has been reported in NETs [36]; in our study, no difference was seen in *SSTR2* expression among primary and metastatic tumors. Of note, both cases of *IDH2mut* ONB were classified as basal, as suggested previously [8], and showed low *SSTR2* expression. We observed an enrichment in genes involved in proliferation pathways, including E2F targets and the G2M checkpoint in the high *SSTR2*-expressing ONB subgroup. Furthermore, *SSTR2* gene expression in the ONB tumor samples was considerably higher than that in a cohort of gastrointestinal NET tumor samples, for which the SSTR2-directed radioactive Lutetium (^177^Lu-DOTATATE) is a U.S. Food and Drug Administration-approved therapy.

In patients with urological cancers and melanoma, SSTR2 expression by IHC has been reported as positively correlated with the response to a checkpoint blockade, suggesting SSTR2 as a potential surrogate marker to identify responders to immunotherapy. An analysis of the TCGA database revealed higher T cell infiltration and enriched inflammatory pathway signatures in high *SSTR2*-expressing tumors [37]. We did not see any significant association between *SSTR2* expression and immune biomarkers in ONB, including TCI score and PD-L1 positivity; however, we observed an increased estimated proportion of antigen-presenting cells and NK cells in high *SSTR2*-expressing ONB. Increased NK-cell infiltration in the tumor microenvironment has been associated with improved clinical outcomes [38,39] and is potentially actionable through NK-targeting immunotherapy agents, including bispecific engagers, thus suggesting treatment options in cancers lacking T cell infiltration. Somatostatin receptor 2-high ONB also showed enrichment in the IFN-α response pathway and mTORC1 signaling, a pathway relevant in NK cell function and activation [40].

In our study, the SNUC cohort had a higher proportion of T cell-inflamed tumors compared to ONB and SNEC, which may translate into a greater susceptibility to immunotherapy. The expression of *SSTR2* was not prominent in SNUC, which is in line with previous findings [17,18], and a moderate correlation between *SSTR2* expression and the infiltration of B and dendritic cells was observed. Similarly, despite the undisputed neuroendocrine differentiation of SNEC, we found comparatively lower *SSTR2* expression, reflective of the conflicting data for SSTR2 staining by IHC in this setting [17]. Thus, an analysis of larger groups across both primary and metastatic samples is needed to fully elucidate SSTR2 expression in SNEC. We found no cases of T cell-inflamed SNEC [29] but observed an intra-tumoral infiltrate of B and NK cells, which warrants further functional analysis.

The expression of *SSTR2* can be exploited for targeted therapies, as shown with the approval of ^177^Lu-DOTATATE (Lutathera) in NETs [41]. Histone deacetylase inhibitors and several chemotherapy agents have been shown to restore *SSTR2* expression, thus enhancing the efficacy of ^177^Lu-DOTATATE therapy in metastatic NET models [42,43,44], and combinational approaches with SSTR2-targeted therapy are currently being investigated. Poly (ADP-ribose) polymerase inhibitors have also been shown to increase PRRT-mediated cytotoxicity [45] and are currently being investigated in clinical trials in combination with ^177^Lu-DOTATATE (NCT05870423). The role of *SSTR2* expression in the setting of immunotherapy is unclear. The combination of PRRT, including SSTR2-directed agents, with CPI has been suggested to result in synergistic action [46]. In NETs, while immunotherapy alone has demonstrated limited activity, radiopharmaceuticals have been shown to increase subsequent CPI efficacy by favoring cellular-mediated killing and promoting a proinflammatory environment [47,48]. Responses to PRRT combined with CPI have been documented in patients with NET with a prior history of failure to CPI alone [49]. Given the high expression of *SSTR2* in ONB, the combinatorial approach of SSTR2-targeted PRRT and CPI merits investigation.

The limitations of our study include the small sample size and the lack of detailed clinical information, including the tumor grade for ONB (sinonasal carcinomas are, by definition, high-grade tumors). Furthermore, bulk RNA-Seq necessitates the use of deconvolution methods to estimate tumor immune infiltration instead of a direct study with single-cell RNA-Seq. Finally, no IHC staining of the SSTR2 protein was available to examine the correlation with *SSTR2* gene expression.

## 5. Conclusions

In conclusion, we showed that *SSTR2* gene expression is prominent in ONB, especially in the neural subtype, and is present, albeit at a lower level, in SNUC and SNEC. We confirmed the immunologically cold microenvironment of SNEC, whereas SNUC appeared to be more frequently associated with a PD-L1 positive and T cell-inflamed phenotype, potentially suggesting a role for immunotherapy-based treatment. In ONB, despite most cases showing a low TCI score with little T cell infiltration, variability in immune infiltrates and occasional tumors with high or intermediate TCI scores demonstrate the necessity to evaluate treatment options on an individualized basis. Further research is needed to determine the utility of SSTR2-directed therapeutic approaches in these rare sinonasal tumors.

## Figures and Tables

**Table 1:** Patient and tumor characteristics.

	ONB	SNUC	SNEC	*p*
Sample Size	N = 26	N = 13	N = 8	
**Age**				
Median age at diagnosis—years, (range)	59 (37–78)	54 (20–76)	61 (35–76)	0.35
**Sex**				
Male N (%)	13 (50.0%)	5 (38.5%)	5 (62.5%)	0.62
Female N (%)	13 (50.0%)	8 (61.5%)	3 (37.5%)	
**Tumor site**				
Metastatic site	8 (30.8%)	10 (76.9%)	6 (75.0%)	0.03
Primary tumor	15 (57.7%)	2 (15.4%)	2 (25.0%)	
Unknown	3 (11.5%)	1 (7.7%)	0 (0.0%)	

Abbreviations: ONB—olfactory neuroblastoma; SNUC—sinonasal undifferentiated carcinoma; SNEC—sinonasal neuroendocrine carcinoma.

## Data Availability

The datasets analyzed during the current study are not publicly available but are available from the corresponding author upon reasonable request.

## Supplementary Materials

The following supporting information can be downloaded at: https://www.mdpi.com/article/10.3390/cancers16233931/s1, Supplementary Figure S1: Heatmap showing *SSTR2* expression (transcripts per million: TPM), prevalence of T cell-inflamed subtypes, prevalence of immune biomarkers, and % immune infiltrate (derived from bulk RNA sequencing using quantTIseq) for ONB tumors with greater than (high) or less than (low) median expression of *SSTR2*. Asterisk indicates statistical significance; *p* < 0.05. Supplementary Figure S2: Unsupervised clustering with genes from Classe et al. [8] depicting genes implicated in KEGG pathways that were more significantly enriched in neural ONB subtype than in basal ONB subtype. Supplementary Figure S3: Overall survival in ONB stratified by *SSTR2* expression. Table S1. Estimated proportion of immune infiltrate in ONB, SNUC, and SNEC tumor microenvironments. Table S2. Estimated proportion of immune infiltrate in ONB stratified by SSTR2 expression.
