# Supplementary material for: Characterization of Somatostatin Receptor 2 Gene Expression and Immune Landscape in Sinonasal Malignancies
Source: Cancers (Basel). 2024 Nov 24;16(23):3931. doi: 10.3390/cancers16233931 (PMC11640466; doi:10.3390/cancers16233931)
Supplement: Supplementary file 1 [file cancers-16-03931-s001.zip › cancers-3302833-supplementary.pdf]

cancers

MDPI

Supplementary Materials

Table S1 – Estimated proportion of Immune Infiltrate in ONB, SNUC and SNEC tumor microenvironment

Median infiltrate (%)	ONB	SNUC	SNEC
B cells	7.07	9.20	8.76
Macrophages M1	0.41	2.99	0.58
Macrophages M2	2.97	3.70	2.44
Monocytes	0.00	0.00	0.00
Neutrophils	0.00	0.48	0.00
NK cells	8.60	3.99	3.99
$CD4^+$ T cells	3.85	0.00	0.00
$CD8^+$ T cells	0.50	0.98	0.00
T regs	0.00	2.39	0.00
Dendritic cells	5.74	4.85	3.01

Table S2 – Estimated proportion of Immune Infiltrate in ONB stratified by *SSTR2* expression

Median infiltrate (%)	*SSTR2*-high ONB	*SSTR2*-low ONB	*p*
B cells	7.05	7.650	ns
Macrophages M1	0.18	0.82	ns
Macrophages M2	4.54	2.77	ns
Monocytes	0	0	ns
Neutrophils	0	0	ns
NK cells	12.96	6.3	0.006
$CD4^{+}$ T cells	4.97	2.19	ns
$CD8^{+}$ T cells	0.5	0.89	ns
T regs	0	0.1	ns
Dendritic cells	7.24	4.98	0.018

Supplementary Figure S1: Heatmap showing *SSTR2* expression (transcripts per million: TPM), prevalence of T cell-inflamed subtypes, prevalence of immune biomarkers and % immune infiltrate (derived from bulk RNA sequencing using quantTIseq) for ONB tumors with greater than (high) or less than (low) median expression of *SSTR2*. Asterisk indicates statistical significance, $p < 0.05$.

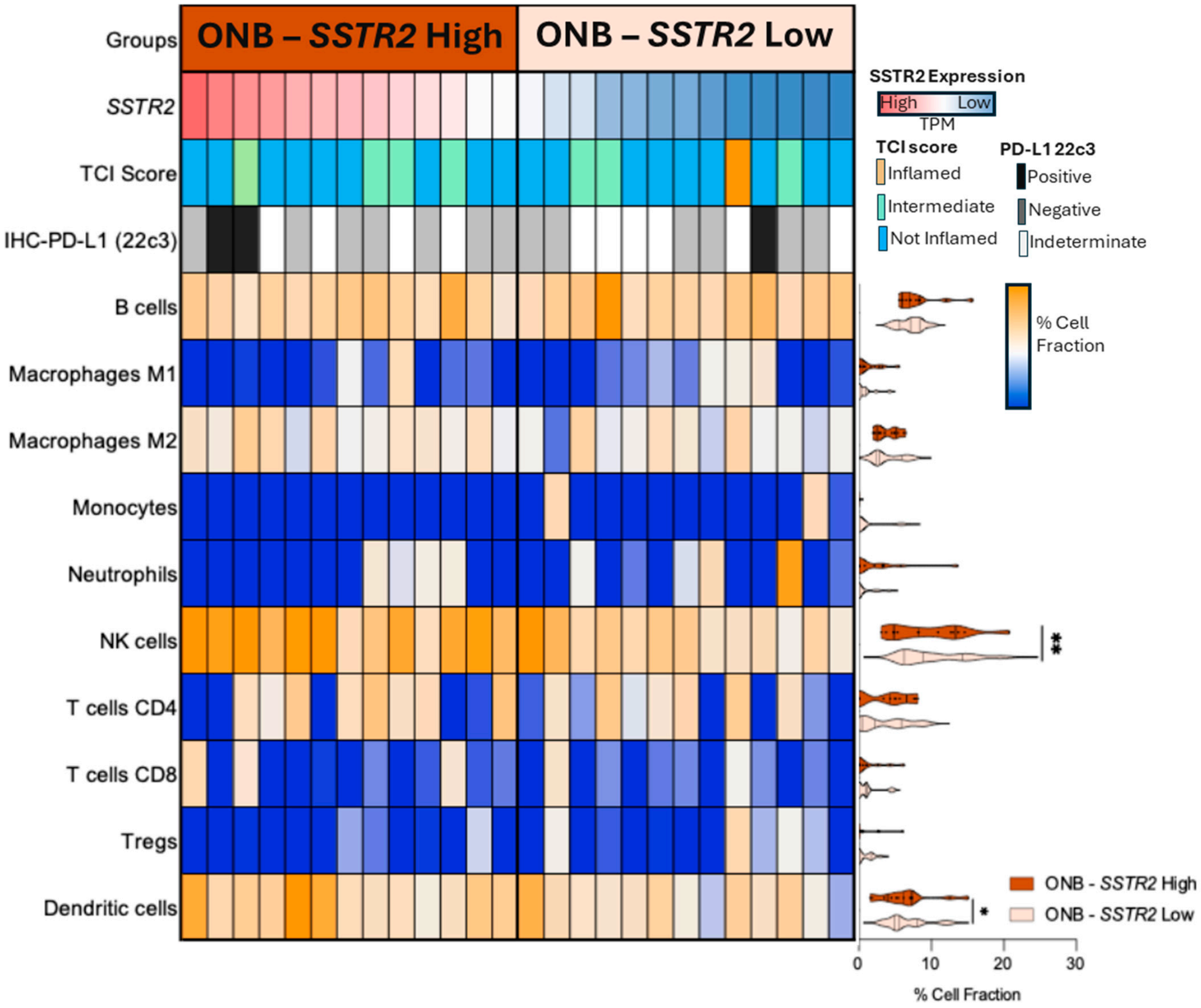

Supplementary Figure S2: Unsupervised clustering with genes from Classe et al. [1] depicting genes implicated in the KEGG pathways that were more significantly enriched in the neural ONB subtype than in basal ONB subtype.

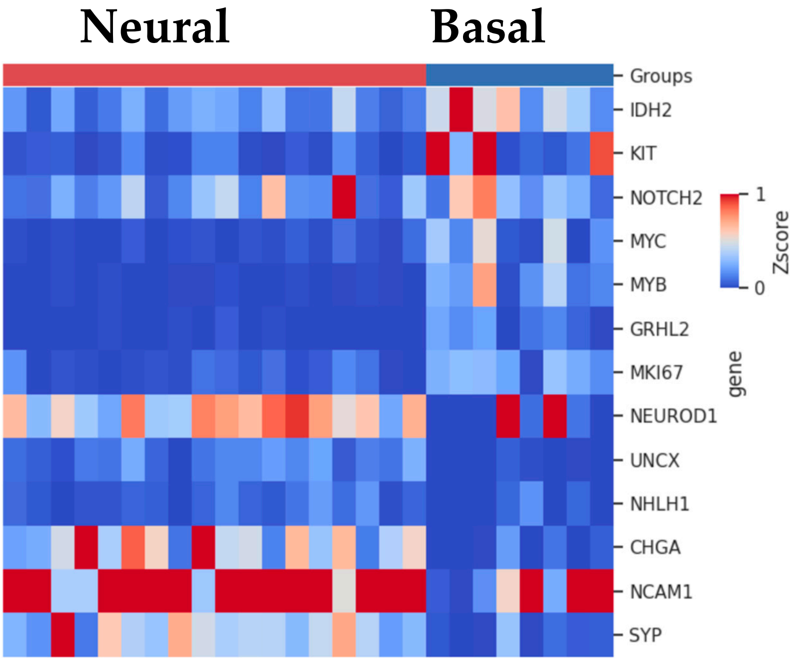

Supplementary Figure S3: Overall survival in ONB stratified by *SSTR2* expression

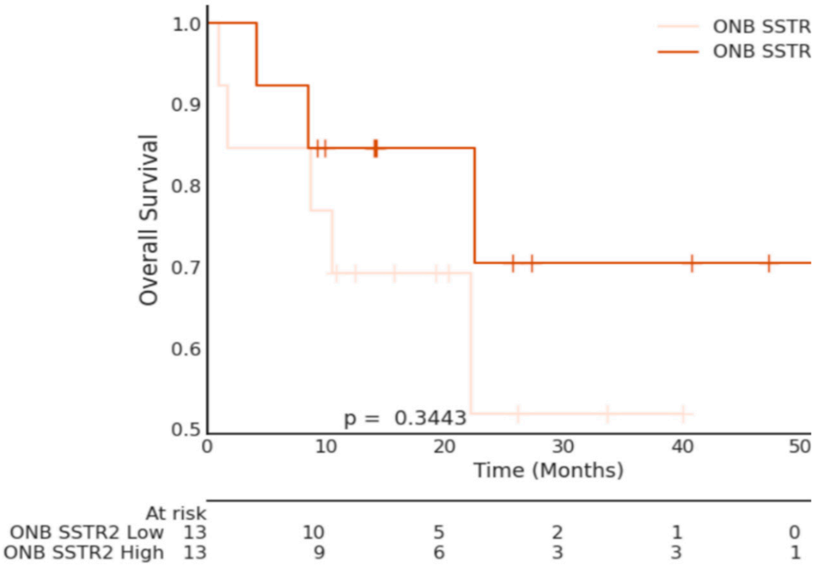

References

1. Classe M, Yao H, Mouawad R, Creighton CJ, Burgess A, Allanic F, Wassef M, Leroy X, Verillaud B, Mortuaire G, et al. Integrated Multi-omic Analysis of Esthesioneuroblastomas Identifies Two Subgroups Linked to Cell Ontogeny. Cell Rep. 2018 Oct 16;25(3):811-21 e5. PMID: 30332658. doi: 10.1016/j.celrep.2018.09.047.
